# Barley Nodulin 26-like intrinsic protein permeates water, metalloids, saccharides, and ion pairs due to structural plasticity and diversification

**DOI:** 10.1016/j.jbc.2023.105410

**Published:** 2023-10-31

**Authors:** Akshayaa Venkataraghavan, Julian G. Schwerdt, Stephen D. Tyerman, Maria Hrmova

**Affiliations:** School of Agriculture, Food and Wine, and Waite Research Institute, Waite Research Precinct, University of Adelaide, Glen Osmond, South Australia, Australia

**Keywords:** 3D molecular modeling of aquaporins, heterologous protein expression, phylogenomics, solute permeation selectivity, stopped-flow spectrophotometry

## Abstract

Aquaporins can facilitate the passive movement of water, small polar molecules, and some ions. Here, we examined solute selectivity for the barley Nodulin 26-like Intrinsic Protein (HvNIP2;1) embedded in liposomes and examined through stopped-flow light scattering spectrophotometry and *Xenopus laevis* oocyte swelling assays. We found that HvNIP2;1 permeates water, boric and germanic acids, sucrose, and lactose but not d-glucose or d-fructose. Other saccharides, such as neutral (d-mannose, d-galactose, d-xylose, d-mannoheptaose) and charged (N-acetyl d-glucosamine, d-glucosamine, d-glucuronic acid) aldoses, disaccharides (cellobiose, gentiobiose, trehalose), trisaccharide raffinose, and urea, glycerol, and acyclic polyols, were permeated to a much lower extent. We observed apparent permeation of hydrated KCl and MgSO_4_ ions, while CH_3_COONa and NaNO_3_ permeated at significantly lower rates. Our experiments with boric acid and sucrose revealed no apparent interaction between solutes when permeated together, and AgNO_3_ or H[AuCl_4_] blocked the permeation of all solutes. Docking of sucrose in HvNIP2;1 and spinach water-selective SoPIP2;1 aquaporins revealed the structural basis for sucrose permeation in HvNIP2;1 but not in SoPIP2;1, and defined key residues interacting with this permeant. In a biological context, sucrose transport could constitute a novel element of plant saccharide-transporting machinery. Phylogenomic analyses of 164 Viridiplantae and 2993 Archaean, bacterial, fungal, and Metazoan aquaporins rationalized solute poly-selectivity in NIP3 sub-clade entries and suggested that they diversified from other sub-clades to acquire a unique specificity of saccharide transporters. Solute specificity definition in NIP aquaporins could inspire developing plants for food production.

Aquaporins (AQPs) are fundamental to water and other solute movements in nearly all living organisms. AQPs are membrane proteins classified in the family of major intrinsic proteins (MIPs) that transport water and neutral solutes (1, 2), although the repertoire of permeated solutes has recently expanded as some AQPs may also transport ions (3). The AQP family consists of seven sub-families of plasma membrane intrinsic proteins (PIPs), tonoplast intrinsic proteins (TIPs), Nodulin 26-like Intrinsic Proteins (NIPs), and small and basic intrinsic proteins (SIPs). Other less common sub-families include Solanaceae X-intrinsic proteins (XIPs) (4) found in fungi, slime molds, and dicots, while GlpF-like intrinsic proteins (GIPs) (5) and hybrid (between PIPs and TIPs) intrinsic proteins (HIPs) are found in green algae, the moss *Physcomitrella* and lycopods (4). AQPs from each sub-family differ in physicochemical properties that underlie their complex roles in osmotic homeostasis, root water uptake from the soil, root, and leaf hydraulic conductance, lateral root emergence, motor cell movement, internode elongation, the diurnal regulation of leaf movements, petal development, biotic interactions, signaling and stomatal dynamics (3, 6, 7).

NIPs, named after soybean nodulin 26 (NOD26), an abundant protein in the peribacteroid membrane of nitrogen-fixing root nodules, are functional equivalents of aquaglyceroporins (8) and occur in barley (9), rice (10), wheat (11), and other plants. Besides water and boric acid (BA) (9, 10), NIPs transport other hydroxylated metalloids such as arsenious and germanic acid (9, 12, 13, 14, 15), glycerol (12), urea (16), hydrogen peroxide (13), acyclic polyols, purines and pyrimidines (17), neutral lactic acid (18), selenious and antimonious acids (19), and aluminum malate (20). Some AQPs facilitate gas diffusions such as CO_2_ (21), NH_3_ (22, 23, 24), and O_2_ (25). When NOD26 was incorporated in lipid bilayers, ionic currents were detected with slight anion over cation selectivity (26, 27). From an evolutionary point of view, plant NIPs (28) and Solanaceae XIPs (“metalloido-porins”) (29), permeating heavy metals and H_2_O_2_ but not water (30), were shown to evolve from cyanobacteria (31) some 1500 million years ago. Then a primordial AQP NIP-like (*aqpN*) gene was acquired by some plants *via* horizontal transfer shedding light on NIPs solute evolution and selectivity (32, 33).

The structural monomeric sub-unit of functional AQP folds into six tilted membrane-spanning α-helices and two short re-entrant α-helices, running in two repeats, with five interconnecting loops forming a right-handed α-helical bundle. A permeating pore running through each monomer has a defined morphology and width in each AQP sub-family; for example, barley HvNIP2;1 has a wide pore along its entire length compared to PIPs and TIPs (2, 34, 35). In native environments, AQPs oligomerize into tetramers along a four-fold rotational axis and create a tightly fitted trapezoid that at structural and functional levels may form an additional central fifth pore. Each AQP monomer has its solute path and with a central pore, the tetramer potentially offers five permeating routes (3, 36). The broad solute selectivity of the monomer pore of NIPs is supported by Asn-Pro-Ala (NPA) motifs that are separated by 4 Å to 5 Å from the aromatic/R selectivity filter residues (2, 36).

To achieve the constant adjustment of water and solute permeability in fluctuating environments, cells developed multiple controls of AQP function through structural features. For example, extended N- and C-terminal regions in NIPs (9, 37) could impact transport, gating, and *in vivo* AQP expression levels. These regions and cytosolic loops (*e.g.*, loop D) in SoPIP2;1 (PDB-Protein Data Bank 2B5F) (38, 39), AtPIP2;4 (PDB 6qim) (40), AtTIP2;1 (PDB 5i32) (41) and recently elucidated rice OsNIP2;1 (PDB 7cjs and 7nl4) (42, 43) have similar spatial dispositions suggesting that dynamics of PIPs, TIPs, and NIPs could be similar. In SoPIP2;1 loop D alongside His193 and dephosphorylated Ser residues trigger pore closure and other structural re-arrangements (38).

To understand the function of HvNIP2;1 in the context of metalloid toxicity, it is fundamental to understand that this trait is a major problem in cereal crops around the world (44). Although metalloids boron and silicon are essential micronutrients, other metalloids such as arsenic and germanium are toxic at certain concentrations, which together with high soil boron pose risks to populations (45). Metalloids exist in soils in the form of amphoteric oxides with atomic radii between 3.43 Å to 4.48 Å, and these differences could be exploited to engineer mutants with selective permeation properties (19).

In the present study, solute selectivity is explored in HvNIP2;1 solubilized from *Pichia pastoris* membranes through embedding in liposomes. The selectivity of HvNIP2;1 towards neutral solutes and ion pairs is examined, establishing that this AQP permeated water, BA, germanic acid, sucrose, and MgSO_4_ and KCl ion pairs at relatively high rates, but also permeated at low rates acyclic polyols, urea and glycerol, other ion pairs, and some mono- and disaccharides of various stereo chemistries. Molecular docking was performed on the monomeric HvNIP2;1 3D AlphaFold 3D model (accession AF-D8V828) to provide molecular-level descriptions of permeation, including identifying structural elements that underlie solute poly-specificity. These data are compared to those of water-selective SoPIP2;1 AQP in an open state conformation (38, 39). Structural data suggested that the wider pore of HvNIP2;1 lined with acidic and aromatic residues, and high pore flexibility, were the key features supporting the permeation of a wide range of solutes. These data were rationalized by phylogenomic analyses of 3157 Viridiplantae (green algae and land plants), Archaean, bacterial, fungal, and Metazoan AQPs to show that the members of the NIP3 sub-clade evolved a unique solute specificity of saccharide transporters through neo-functionalization after the emergence of tracheophytes. The HvNIP2;1 substrate poly-specificity definition underscores yet unidentified roles of this AQP in plant growth, metabolism, and development.

## Results

### Cloning, expression, solubilization, purification, and liposomal reconstitution of HvNIP2;1

**Figure 1:**
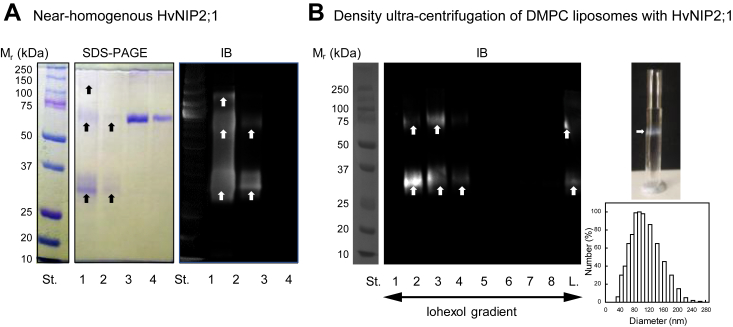
**SDS-PAGE and immunoblot (IB) analyses of near-homogenous HvNIP2;1 purified by IMAC, and density gradient ultra-centrifugation of DMPC liposomes with reconstituted HvNIP2;1.***A*, monomeric (apparent molecular mass 34 kDa), dimeric (70 kD), and tetrameric (150 kDa) forms of HvNIP2;1 (lanes one and 2) are indicated by *arrows* at approximately 0.5 and 1 μg protein loadings, respectively. Lanes three and four contain BSA (Fraction V) at one or 2 μg loadings, respectively. Immunoblot (IB) analysis proceeded with anti-His antibody, as described in Experimental procedures. St. lanes indicate molecular masses of protein standards. *B*, density gradient ultra-centrifugation of DMPC liposomes with embedded HvNIP2;1 and proteo-liposomal sizing. Lanes 1 to 8 are fractions collected after ultra-centrifugation in the iohexol gradient, where L. (lane 9) contains non-fractionated preparation. Lane with standard proteins (St.) indicates molecular masses of protein standards. HvNIP2;1 was detected by IB with an anti-His antibody. Right top panel shows the test tube of liposomes with reconstituted HvNIP2;1 (*arrow*) after the iohexol gradient floating, forming a white diffuse band. Bottom image displays the size distribution profile of proteo-liposomes analysed by the NICOMP 380 Particle Sizing System, as described in Experimental procedures.

The native sequence of HvNIP2;1 was cloned into the *HvNIP2;1*-Myc-6xHis-pPICZ-B DNA fusion (46, 47, 48) and expressed in *P. pastoris*. *Pichia* transformants were screened for high-level protein expression, where HvNIP2;1 appeared predominantly in alkaline soluble or insoluble alkaline/urea fractions (data not shown). HvNIP2;1 was solubilized in styrene-maleic anhydride co-polymer 3:1 (SMA) (49) and purified by Immobilized Metal Affinity Chromatography (IMAC) (46, 47). Near-homogenous HvNIP2;1 (Fig. 1*A*) was reconstituted in 1, 2-dimyristoyl-sn-glycero-3-phosphocholine (DMPC) liposomes that were floated on an iohexol (Accudenz) gradient (50, 51) to obtain homogenous populations of proteo-liposomes of around 100 μm in size (Fig. 1*B*) used to define permeation properties of HvNIP2;1.

The identity of purified HvNIP2;1 confirmed by immunoblot, SDS-PAGE, and IB analyses using anti-HvNIP2;1 antibody (Fig. 1) revealed that HvNIP2;1 folded predominantly into monomeric (apparent molecular mass of around 34 kDa) and dimeric (apparent molecular mass of around 70 kDa) forms, but tetrameric forms (apparent molecular mass of around 150 kDa) were also detected (Fig. 1*A*). The inspection of the tetrameric model of HvNIP2;1, built on the coordinates of OsNIP2;1 (43) (the structural aspects of the model discussed below), indicated that the four monomers displayed extended hydrophobic interactions, which included phenylalanine, leucine and valine residues that linked individual subunits. These types of interactions were noted with other aquaporins (38, 42, 43).

Electrophoretic profiles of purified HvNIP2;1 in the form of diffused bands suggested that HvNIP2;1 was N-glycosylated at putative S26 although O-glycosylation sites could also be occupied. No degradation of purified HvNIP2;1 was observed after 3 weeks at 4 °C indicating that it was amenable for permeation studies. To confirm that HvNIP2;1 was incorporated in DMPC liposomes and not simply aggregated during HvNIP2;1 reconstitution, liposomes with embedded HvNIP2;1 were subjected to floatation by iohexol density gradient ultra-centrifugation. As indicated, fractions 2 to 4 containing proteo-liposomes with HvNIP2;1, detected with anti-His antibody, floated near the top of the gradient, and most of HvNIP2;1 incorporated in DMPC liposomes (Fig. 1*B*).

### Permeation properties of HvNIP2;1

**Figure 2:**
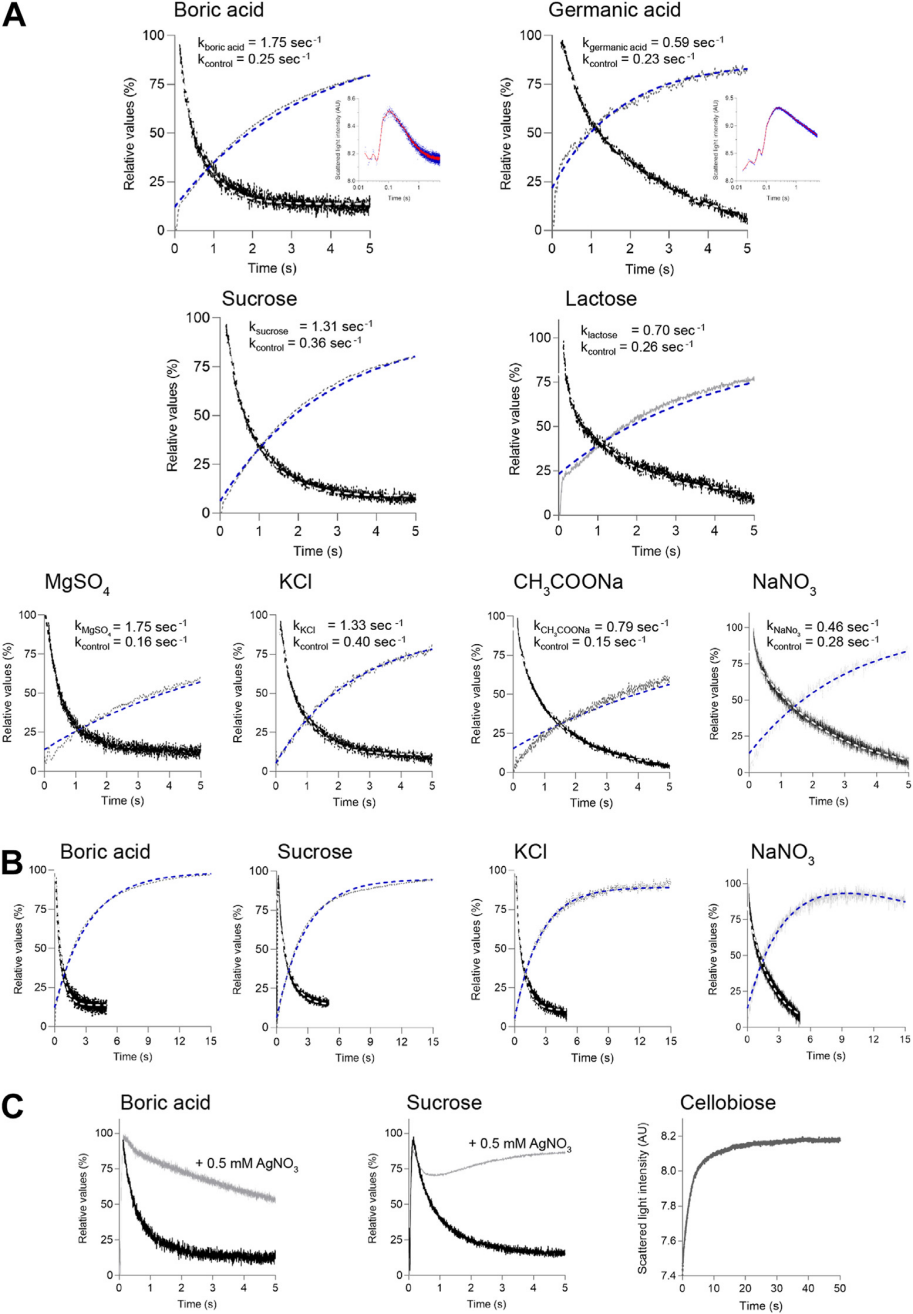
**Transport of permeants by HvNIP2;1 embedded in liposomes.***A–C*, HvNIP2;1 solubilized by SMA and reconstituted in DMPC liposomes were exposed to gradients of solutes generating osmotic gradients with BA and germanic acid (*top panels*), where insets show full profiles (shown also in Fig. S1), disaccharides sucrose and lactose (*bottom-middle panels*), and MgSO_4_, KCl, CH_3_COONa, and NaNO_3_ (*bottom panels*). The uptake of permeants was measured by stopped-flow spectrophotometry in the liposome buffer] at the concentration of 0.2 M (340 mOsmol/kg). Fast water flow (shrinkage of liposomes) is shown by relative values at less than 0.1 s that approach 100% (the real values indicated in Fig. S1 and S2). The steep downward slopes of light scattering traces after this time indicate swelling slopes of HvNIP2;1 proteo-liposomes, where *black curves* indicate normalised data (as relative values) and *white dashed curves* represent non-linearly fitted data using a one-phase decay model. Light scattering traces of control liposomes (lacking HvNIP2;1) are drawn in *grey* for the first five (panels *A*) and 15 s (panels *B*), and non-linear fits using a one-phase association model are shown in *blue dashed curves*. 0.5 mM AgNO_3_ was used to inhibit transport in HvNIP2;1 (panels *C*; *grey curves*). In panel *A*, the calculated rate constants for each combination of permeants are indicated in s^−1^ for liposomes with HvNIP2;1 and control liposomes lacking HvNIP2;1. Plots in panels *B* (control liposomes and proteo-liposomes for BA, sucrose, KCl, and NaNO_3_) and panel *C* (right plot – data of control liposomes exposed to cellobiose) indicate that no solute leakage was observed; the same non-solute leakage profiles were observed for control liposomes. Data were plotted in relative values (%) in GraphPad Prism 9.

Measurements of water and solute permeability, based on osmotic gradients generated by co-incubating DMPC liposomes with embedded HvNIP2;1 or control liposomes lacking the protein, and osmolytes at high concentrations, were assessed by stopped-flow light scattering spectrophotometry (Figs. 2, S1 and S2 and Table S1). These co-incubations created an osmotic gradient that in the first phase led to increased rates of water efflux causing the shrinkage of liposomes, while in the second phase, proteo-liposomes increased their volumes and swelled due to rate-limiting solute transport of water (52). Thus, as shown in Figure 2, fast water flow (shrinkage of liposomes) is reflected by relative values at less than 0.1 s that approach 100% (real values are presented in Figs. S1 and S2). Conversely, the steep downward slopes of light scattering traces after this time then indicate swelling slopes of HvNIP2;1 proteo-liposomes, where black and white curves indicate normalized and non-linearly fitted data using a one-phase decay model. This occurred with liposomes with reconstituted HvNIP2;1 that mediated solute permeation, but not for control liposomes or when liposomes with embedded HvNIP2;1 were incubated with 0.5 mM AgNO_3_, a potent blocker of AQPs (53), for which a re-swelling phase was incomplete (Fig. 1*C*). HvNIP2;1 permeated BA and germanic acid at higher rates, while glycerol, d-mannitol and d-sorbitol, and urea were permeated at low rates (Figs. 2, S1 and S2 and Table S1). Surprisingly, HvNIP2;1 permeated at high rates the disaccharide sucrose, as previously observed (46), and some monosaccharides (d-xylopyranose, l-arabinofuranose), and hydrated ion pairs KCl and MgSO_4_, while lactose, the CH_3_COONa, and NaNO_3_ ion pairs were transported at lower rates. Conversely, NaF, the neutral monosaccharides d-glucopyranose, d-fructopyranose, d-galactopyranose, d-mannopyranose, and d-mannopyranopheptaose, charged monosaccharides d-glucosamine, N-acetyl β-d-glucosamine and d-glucuronic acid, disaccharides trehalose, cellobiose and gentiobiose, and trisaccharide raffinose were permeated at even lower rates (Fig. S2). Permeation rate constants and derived permeability coefficients (*P*) (Table S1) for water, metalloids, saccharides, and other solutes, and ion pairs descended in these orders:

Water and metalloids: H_2_O >> BA > germanic acid.

Polyols and urea: urea > glycerol > d-mannitol > d-sorbitol.

Saccharides: sucrose > l-arabinofuranose > lactose > d-glucose > d-fructose.

Ion pairs: MgSO_4_ > KCl > CH_3_COONa > NaNO_3_.

All permeants: H_2_O >> MgSO_4_ ≥ BA > KCl ≥ sucrose > urea > glycerol > CH_3_COONa > d-mannitol ≥ lactose > germanic acid > NaNO_3_ > d-sorbitol > d-glucose > d-fructose.

**Figure 3:**
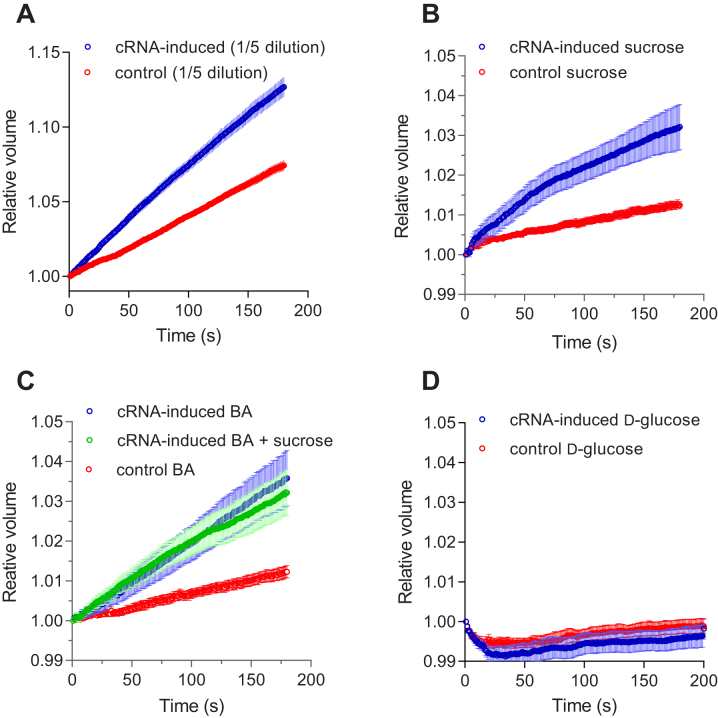
**Uptake of permeants by *Xenopus laevis oocytes* transformed with cRNA of HvNIP2;1 and by water-injected (non-transformed) oocytes.***A*, an increase in relative volume (V/V_0_) over time of transformed oocytes after replacing incubation solutions with 5-fold diluted ND96 (n = 7 oocytes). *B*, isosmotic solutions supplemented with 160 mM sucrose (n = 7 oocytes), *C*, 160 mM BA and 80 mM BA with 80 mM sucrose added together (n= 7 oocytes), and *D*, 160 mM d-glucose (n = 8 oocytes). Controls (n = 7–eight oocytes) refer to water-injected (non-transformed) oocytes. Three batches of oocytes showed the same results. Error bars represent Standard Error of Measurements.

Measurements of water and solute permeability for selected solutes by HvNIP2;1 were confirmed with *Xenopus laevis* oocytes that were injected with cRNA of HvNIP2;1 or water as controls (Fig. 3), where the swelling was measured in response to water gradient (Fig. 3*A*) confirming water permeation by HvNIP2;1, and isosmotic BA, sucrose, and d-glucose concentration gradients (Fig. 3). Transport of BA and sucrose was observed (Fig. 3, *B* and *C*), while d-glucose permeation did not occur (Fig. 3*D*), as also shown using the stopped-flow light scattering analyses (Fig. S2). No detectable interaction was seen between BA and sucrose, when permeated together (Fig. 3*C*). In oocytes, incubated with 0.5 mM H[AuCl_4_] (53), which is the known histidine and thiol groups modifier, solute transport was inhibited.

### Molecular model of HvNIP2;1 and the SoPIP2;1 crystal structure, and docking of sucrose

**Figure 4:**
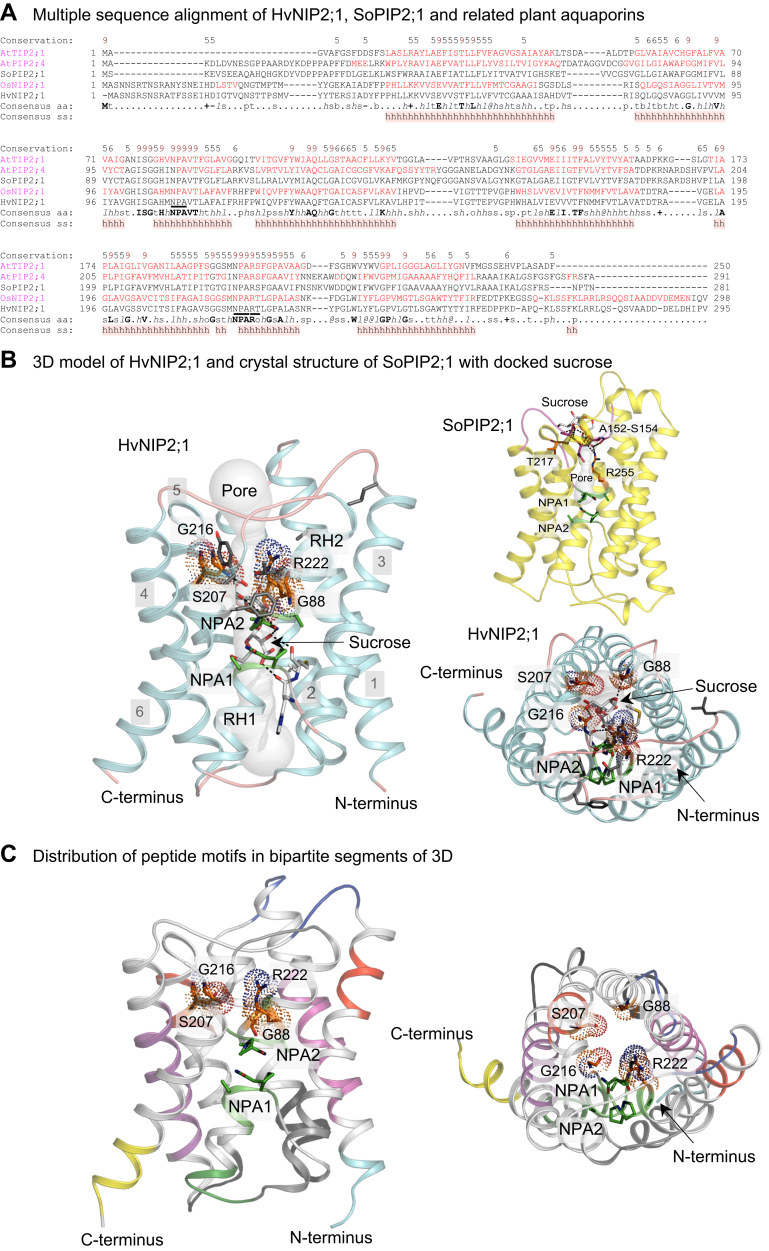
**Structural model of HvNIP2;1 and the crystal structureof SoPIP2;1.***A*, Multiple sequence alignment of HvNIP2;1 and SoPIP2;1 (PDB-Protein Data Bank 2B5F), and plant structural homologues AtTIP2;1 (PDB 5i32), AtPIP2;4 (PDB 6qim), and OsNIP2;1 (PDB 7cjs and 7nl4). Sequences were aligned using ProMals3D (91), where the alignment indicates the level of conservation of residues (absolutely conserved residues on the scale 5–9 in brown and bold). Respective indices ‘s’, ‘p’, ‘l’ and ‘h’ indicate small (A, G, C, S, V, N, D, T, P), polar (D, E, H, K, N, Q, R, S, T), aliphatic (I, V, L) and hydrophobic (W, F, Y, M, L, I, V, A, C, T, H) residues. Consensus amino acid residues (aa) and secondary structure elements (ss) are shown in three diversified aquaporin groups (*magenta*). Some of the key structural elements and residues for HvNIP2;1, such as NPA motifs, conserved R222 and Froger’s P2 (T223), indicated in panel *B* and Table S2, are marked by *black bars*. *B*, Cartoon representation of monomeric HvNIP2;1 (*cyan cartoon*) in two orthogonal orientations (rotated by approximately 90°) features a predicted pore (cpk spheres; pore radii equal to sphere diameters) that contains the docked sucrose (cpk sticks). Separations (*dashed lines*) of sucrose with surrounding residues are between 2.4 Å and 3.2 Å. Selectivity filter residues G88, S207, G216, and R222 (*orange sticks with dots*) are near the pore entrance, while the NPA1 and NPA2 motifs (*green sticks*) are located in the pore. Residues in the Froger’s P1-P5 positions (L148, T223, A227, Y239, and F240) are shown in *grey sticks*. Numbering of α-helices 1 to 6 and re-entrant α-helices (RH1 and RH2) are indicated. The crystal structure of SoPIP2;1 (*yellow cartoon*) with the predicted pore, NPA motifs and the docked sucrose molecule is shown with interacting residues (A152, N153, S153, T217, R255) at the distances between 2.2 Å and 3.5 Å (*dashed lines*). *C*, the bipartite symmetry distribution of peptide motifs in two orthogonal orientations (rotated by approximately 90°). Motif pairs and their positions in secondary structural elements are coloured identically in each half of the structure. Selectivity filter residues G88, S207, G216, and R222 (*orange sticks with dots*), residues in NPA1 and NPA2 motifs (*green sticks*) and in the Froger’s P1-P5 positions are indicated. Six N- and 20 C-terminal residues are omitted for clarity in panels *A* and *B*.

To investigate the structural features of HvNIP2;1 and work out the structural basis of sucrose permeation, we used the structural bioinformatics approach and aligned the sequences of HvNIP2;1 and SoPIP2;1 (PDB-Protein Data Bank 2B5F), and the plant structural homologs AtTIP2;1 (PDB 5i32), AtPIP2;4 (PDB 6qim), and OsNIP2;1 (PDB 7cjs and 7nl4). As shown in Figure 4*A*, the HvNIP2;1 model has nearly an identical secondary structure distribution to that of the crystal structures of Lsi1 (42) and OsNIP2;1 (43). Here, the distribution of secondary structures, such as α-helices and interconnecting loops coincided (Fig. 4*A*). We also utilised the 3D AlphaFold HvNIP2;1 model (AlphaFold Protein Structure Database accession D8V828; (54)), which features six tilted membrane-spanning α-helices, two short re-entrant α-helices in two repeats, and five interconnecting loops forming an α-helical bundle (Fig. 4*B*; HvNIP2;1 – cyan cartoon). Thus, the respective 0.67 Å and 0.45 Å root-mean-square deviation values between OsNIP2;1 and Lsi1, at the sequence similarity/identity of 74%/68% and 80%/71% (using Needle EMBOSS algorithm; (55)) were unsurprising. In the HvNIP2;1 model, the selectivity filter residues G88, S207, G216, and R222 constituting the GSGR motif were positioned near the entrance to the pore (Fig. 4*B*), while the residues of the two conserved NPA1 and NPA2 motifs (Fig. 4*B*) were located deeper in the pore, in accordance with the positions of these structural elements in both crystal structures. A single solute-conducting pore in HvNIP2;1, investigated through Caver (56) in monomeric HvNIP2;1 was wide alongside its lengths (approximate diameter between 2 Å and 3 Å) without forming constricted regions (approximate diameter range between 2.2 Å and 2.6 Å), and contrary those observed in other AQPs near NPA motifs, such as *e.g.*, in the SoPIP2;1 crystal structure (38, 39) (Fig. 4*B*; SoPIP2;1 – yellow cartoon).

Individual monomers of HvNIP2;1 are expected to form a quaternary assembly, while the disposition of six α-helices followed a pseudo-two-fold axis that run along a membrane normal and was sub-divided an hour-glass fold with bipartite segments. A significant symmetrical distribution of repeating peptide motifs was observed in each bipartite HvNIP2;1 segment (Fig. 4*C* and Table S2) detected by the Multiple EM (Expectation Maximization) for Motif Elicitation (MEME) analysis (57). At least ten peptide motifs were identified in each segment based on *p*-values (Table S2).

To investigate the permeation of sucrose, observed in HvNIP2;1 through stopped-flow light scattering spectrophotometry and oocyte swelling, this disaccharide molecule was docked in HvNIP2;1 (Fig. 4*B*), by means of HDOCK, which is based on a hybrid algorithm of template-based modeling and *ab initio* free docking (58). The docking unambiguously showed that the sucrose can be accommodated in at least three favorable positions in the pore, making interactions with several residues. One such example indicated that sucrose interacted with carbonyl oxygens of M107 and H160 in the vicinity of the NPA1 motif, carbonyl oxygens of G216, and S217 near the NPA2 motif, and ND2 nitrogen of N219 participating in the NPA2 motif (Fig. 4*B*; left and right-bottom panels; HvNIP2;1 – cyan cartoon); these interactions were formed at separations between 2.2 Å and 3.2 Å.

To increase the accuracy of data interpretation with HvNIP2;1, the monomeric high-efficiency water-permeating SoPIP2;1 AQP in an open state conformation (38, 39) was used as an accessory structural target in the docking experiment (Fig. 4*B*; right-top panel; SoPIP2;1 – yellow cartoon). With SoPIP2;1, the sucrose disaccharide could not localise inside the pore (approximate diameter range between 1.0 Å and 2.1 Å) and it was always contained within the surface or at the entry or exit to the pore of the SoPIP2;1 in all examined 100 poses. Here, the prominent GGGANSVALGNYK loop (residues 149–161), was localized at the entry of SoPIP2;1, which obstructed the pore and prevented the passage of the sucrose molecule through the pore. Underneath this loop, the second TGTGINPAR loop (residues 217–225) was identified, consisting of nine residues, which also carried the NPA motif and highly conserved R225. Residues from both loops, more specifically A152, N153 and S154 (first loop) and T217 and R225 (second loop) made close contacts with sucrose at separations between 2.2 Å and 3.5 Å, immobilised it and prevented its passage through the SoPIP2;1 pore (Fig. 4*B*; right top panel). Additionally, it would also be impossible for sucrose to bypass the aromatic/R constriction region point of around 2.1 Å (38), which would be too narrow to accommodate sucrose passage.

### Phylogenomic analyses of MIP proteins

**Figure 5:**
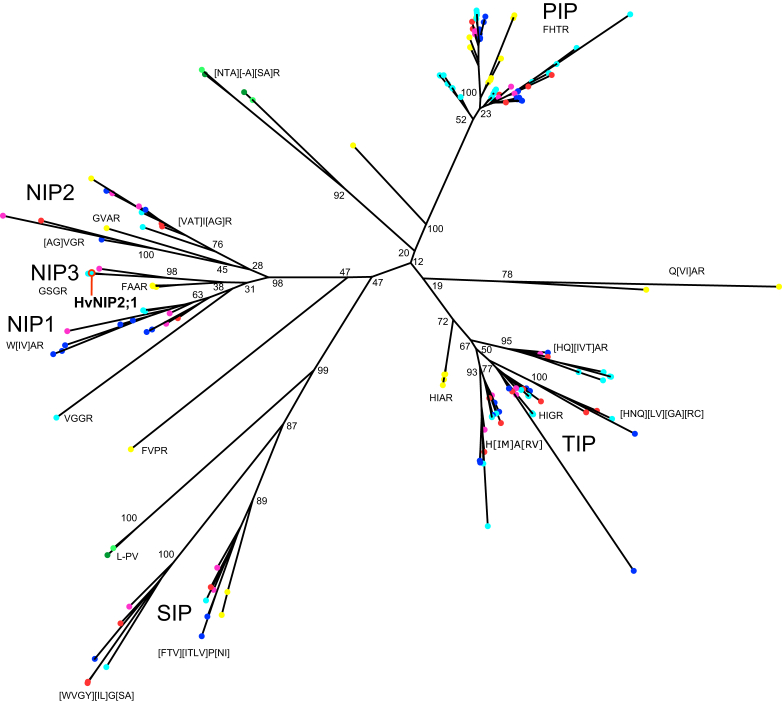
**Evolutionary relationships of Viridiplantae MIP proteins.** RAxML tree of 164 Viridiplantae MIP proteins. Terminal nodes are colour coded by species: *dark green*, *Chlamydomonas reinhardtii*; *light green*, *Volvox carteri*; *yellow*, *Physcomitrella patens*; *magenta*, *Amborella trichopoda*; *red*, *Spirodela polyrhiza*; *cyan*, *Hordeum vulgare*; *blue*, *Arabidopsis thaliana*. A *red circle* marks HvNIP2;1 (in bold) investigated in this work. Selectivity filter residues are noted adjacent to relevant clades and residues enclosed in square brackets indicate variations at those positions. Bootstrap support values are indicated at major nodes.

To define the phylogenetic relationships, explicitly the variations in selectivity filter residues of MIPs—which define solute selectivity and thus the function of plant aquaporins—we used Randomized Axelerated Maximum Likelihood (RAxML) phylogenetic analysis as a tool. This widely used approach embodies a fast-maximum likelihood tree search algorithm that reconstructs phylogenetic trees with exceptional likelihood scores. Here, we analyzed 164 Viridiplantae MIP sequences from *Chlamydomonas reinhardtii*, *Volvox carteri*, *Physcomitrella patens*, *Amborella trichopoda*, *Spirodela polyrhiza*, *Arabidopsis thaliana*, and *Hordeum vulgare* (Fig. 5). These analyses indicated that these 164 Viridiplantae (Fig. 5) and additional 2993 archaean, bacterial, fungal, and metazoan entries of the MIP family (Pfam database PF00230) (Fig. S3 and Dataset S1) clustered in the four major TIP, NIP and SIP clades, where the PIP and TIP clades covered the majority of MIP entries.

Barley HvNIP2;1 (Fig. 5; marked in bold) with the GSGR selectivity filter residue signature (that is included in square brackets in Fig. 5) was resolved to form a monophyletic group with *A. thaliana* and *A. trichopoda*. We also indicated that HvNIP2;1 split from *P. patens* that carried the FAAR signature, and that the three NIP1, NIP2, and NIP3 representative clades emerged prior to the evolution of tracheophytes (ferns and seed plants), where the NIP2 and NIP3 clades contained basal *Physcomitrella* sequences, and where a NIP1 representative was perhaps lost. The functional evolution of the NIP clades was evident, and this was indicated by the fact that the selectivity filter in *Physcomitrella* (FAAR) diversified into the GSGR signature, specifically in the NIP3 sub-clade, following the tracheophyte/*Physcomitrella* split. This finding contrasted with the PIP entries, where the FHTR signature was highly conserved in all examined PIP entries (Fig. 5). From the structural point of view, and in accordance with this phylogenomics analysis, the GSGR selectivity filter residue pattern (three smaller side chains with conserved R) in NIP3 entries supports the permeation of larger solutes such as hydroxylated metalloids and certain saccharides (Figs. 2, 3, S1 and S2 and Table S1). Conversely, in PIPs, TIPs, and SIPs, these signatures contained bulky residues. These included aromatic phenylalanine, histidine, tryptophan, tyrosine and other bulky sidechains in the first positions, more specifically: F for the PIPs; His, N, Q, M or I for the TIPs; W, F, Y, V or T for the SIPs, and also W for the NIP1, and V, A or T for the NIP2 clade entries, compared to the NIP3 subclade, where the glycine residue was found (Fig. 5). These selectivity filter residue characteristics agreed with the published data (2, 33, 59, 60, 61, 62), where HvNIP2;1 consistently clustered in the NIP3 sub-clade.

## Discussion

In the present work, we observed that HvNIP2;1 when reconstituted in liposomes, permeated large cyclic saccharide molecules such as sucrose, l-arabinofuranose, and lactose but not d-glucose of d-fructose (Figs. 2, S1 and S2 and Table S1). This is a novel observation not reported previously for any AQP, including other structurally similar NIPs or NOD26. AQP9 from humans and a rat were found to transport large molecules including polyols (glycerol, d-mannitol, d-sorbitol), purines (adenine), pyrimidines (uracil, 5-fluorouracil), and thiourea, but not cyclic saccharides (63, 64). To define the saccharide specificity permeation by HvNIP2;1, an extensive panel of substrates was examined. Here, neutral (d-xylopyranose, d-glucopyranose, d-fructopyranose, d-galactopyranose, d-mannopyranose and d-mannopyranopheptaose) and charged (d-glucosamine, N-acetyl β-d-glucosamine, and d-glucuronic acid) monosaccharides, disaccharides (trehalose, cellobiose, gentiobiose), and the trisaccharide raffinose were permeated at lower rates than sucrose, l-arabinofuranose and lactose (Figs. 2, S1 and S2 and Table S1).

The next group of permeants transported by HvNIP2;1 included KCl and MgSO_4_ ion pairs, while others such as CH_3_COONa and NaNO_3_ were permeated at lower rates (Figs. 2 and S2) and NaF was impermeable (Fig. S2). These observations support previously observed electrogenic ion conductance through NOD26 (structurally similar to HvNIP2;1) when embedded in lipid bilayers (26). Our observations that HvNIP2;1 embedded in liposomes permeated both KCl and MgSO_4_ ion pairs is a new observation for any NIP-type AQP. It remains to be established if this transport also translates to an electrogenic ion conductance as opposed to neutral ion pair permeation. Notably, previous studies described electrogenic ion conductance in human AQP1 and AQP6, AtPIP2;1 (that has different features compared to SoPIP2;1) and AtPIP2;2, HvPIP2;8 and OsPIP1;3, when expressed in oocytes or Human Embryonic Kidney (HEK) 293 cells (65, 66, 67, 68, 69). It was theorized that electrogenic ion conductance could proceed through a central pore, where four monomeric AQPs converge, and that at least in human AQP1 this function could be important for cGMP-mediated gating (36). Nevertheless, neutral ion pair permeation in some AQPs could also occur *via* monomer pores.

HvNIP2;1 embedded in liposomes, also permeated hydroxylated metalloids BA and germanic acid and some other neutral solutes known to be permeated by AQPs (Figs. S1 and S2 and Table S1). Some permeability to BA, urea, and glycerol was observed in control liposomes lacking HvNIP2;1 or in proteo-liposomes incubated with AgNO_3_ (Fig. 2*C*), as these small molecules could permeate passively through lipid bilayers (52, 70). It was observed that the osmotic water permeability coefficient of HvNIP2;1 (*P*_water_ = 3.98 × 10^−2^ cm s^−1^) was 240-fold higher than that of empty liposomes (Table S1) and similar to the liposome-reconstituted *Escherichia coli* water-permeable aquaporin-Z (*P*_water_ = 2.83 × 10^−2^ cm s^−1^) (71) but higher than that of NOD26 (*P*_water_ = 1.2 × 10^−2^ cm s^−1^) (72). The comparison of *P*_*BA*_ = 2.50 × 10^−6^ cm s^−1^ for HvNIP2;1 with *P*_*BA*_ of tapetal NIP7;1 (1.41 × 10^−6^ cm s^−1^) involved in pollen cell wall formation (73), indicated that these values were similar, although in these analyses it is imperative to consider differences in the protein content of liposomes, embedding directionality and protein homogeneity. As for other solutes such as urea, it was surprising but not unexpected that in HvNIP2;1, its permeation rate compared to that for BA, differed (Fig. S1 and Table S1), although their molecular radii are similar (BA – 2.56 Å; urea – 2.30 Å), which agreed with data of rice Lsi1 (74) and soybean NOD26 (75), where low or no urea permeability was reported.

These findings were validated through oocyte swelling with HvNIP2;1 expressed in *X. laevis*, where water, BA, and sucrose transport but not glucose transport occurred (Fig. 3), confirming stopped-flow recordings with HvNIP2;1 in proteo-liposomes, whereas after the application of AgNO_3_ permeability was completely suppressed. Notably, BA and sucrose did not compete during transport when applied together suggesting that they could be co-permeated (Fig. 3).

Sucrose permeation by the 3D AlphaFold model of HvNIP2;1 was confirmed by docking, where HvNIP2;1 contributed with M107, H160, G216, S217, and N219 as pivotal residues (Fig. 4), while no permeation was observed in SoPIP2;1 AQP, also investigated through docking (Fig. 4*B*; right top panel). To explain the inability of HvNIP2;1 to permeate smaller monomeric d-glucose or d-fructose molecules compared to the disaccharide sucrose (a larger molecule) that we observed, we suggest that the voluminous HvNIP2;1 pore is decorated with hydrophobic and hydrophilic residues. We assume that in the HvNIP2;1 pore, hydrophilic glucose could get trapped in pockets, while sucrose, due to its larger size avoids this trapping and slides through the pore. Conversely, a narrower and less capacious SoPIP2;1 would collectively offer an unfavorable milieu for saccharide molecules to pass through.

In planta, HvNIP2;1 AQP is expressed in roots and localized to epidermal and cortical cells of seminal roots and hypodermal cells in lateral roots (9, 10). The biological significance of our key findings of sucrose permeation means that HvNIP2;1 could carry these permeants along with water and metalloids and that this could have profound importance in plant physiology (35, 76). Through this permeation, HvNIP2;1 located on root cell membranes could recover sucrose directed from apoplastic (extracellular) to cellular environments. Consequently, this permeation route could constitute the novel element of a plant’s cellular saccharide-transporting machinery.

To investigate the evolutionary origin of NIP AQPs, the phylogenetic reconstruction of 164 Viridiplantae MIPs was conducted to reveal that PIP, TIP, and SIP clades diversified before the chlorophyte (green algae) and embryophyte (land plants) split. This finding proposes that a single duplication event resulted in SIPs, or that multiple duplication events occurred before the chlorophyte and embryophyte split. These analyses used a relatively complex substitution model avoiding insufficient data for maximum likelihood but still kept accuracy during reconstruction, although there was little support for establishing deep relationships in MIPs as these sequences are relatively short. Hence, we restricted our maximum likelihood analyses to Viridiplantae only (Fig. 5), and separately to Virdiplantae, archaean, bacterial, fungal, and metazoan entries (Fig. S3 and Dataset S1). Notably, we observed increased gene duplication events in angiosperms (flowering plants) in all analyzed clades, our phylogenetic reconstruction showed that there were ancestral gene losses in chlorophytes (green algae), and where deep relationships between the NIP, PIP, and TIP entries were obscured by poor node support (Fig. 5 and Fig. S3).

Notably, and relevantly to the experimental permeation data obtained in this work, the NIP3 sub-clade segregated clearly and carried the GSSR selectivity filter signature. This NIP3 clade was lost or reduced in eudicots, although the deep phylogenetic relationships of the AQP clades were less supported (Fig. S3). Nevertheless, the NIP3 clade segregation suggested that the pore residues were prone to residue alterations (variations) to modulate solute permeation selectivity in a NIP3 sub-clade, which would allow the permeation of hydroxylated metalloids and larger molecules such as sucrose (Fig. 2 and Fig. S1). Meanwhile, the PIP entries positioned on the short molecular branches, showed strong selectivity filter residue conservation in Viridiplantae, while the examined NIP, TIP, and SIP entries underwent more substitutions over time. In agreement with the published data (33, 60, 61), our analysis confirmed that the three NIP1-NIP3 sub-clades diversified early during embryophyte evolution, before the split from the tracheophytes. Further, the NIP3 sub-clade separated from those of NIP1 and NIP2 AQPs or appeared to be lost or reduced in eudicots (Fig. S3). Here, the NIP3 entries showed a clear dichotomy in the signatures of GSSG and FAAR selectivity filter residues (Fig. 5), which ultimately dictates their solute selectivity. This event may have resulted in gaining selectivity that would allow the NIP3 members to permeate specific saccharides, as shown in this work.

In conclusion, our phylogenomic analyses of sequences comprising 3157 AQPs (Figs. 5 and S3) propose that the HvNIP2;1 acquired a unique solute specificity permeation as a saccharide transporter (Figs. 2, 3, S1 and S2 and Table S1) and that its structural model (Fig. 4 and Table S2) supports this concept, which is the leading notion of this work.

## Experimental procedures

Materials and procedures used for fractionation and enzymatic digestion and disruption of *Pichia* cells, urea/alkali treatment of microsomal membrane fractions, solubilization from the urea/alkali-treated microsomal membrane fractions of HvNIP2;1 and other analytical techniques are detailed in Supplementary information.

### Cloning of HvNIP2;1 and *Pichia pastoris* clone selection

Cloning of HvNIP2;1 native cDNA (UniProtKB accession D8V828) in the pPICZ (frame B) (Invitrogen) expression vector yielding the native *HvNIP2;1*-Myc-6xHis-pPICZ-B DNA fusion, destined to be transformed in *P. pastoris*, and *Pichia* clone selection were conducted as described (46, 47, 48).

### Barley HvNIP2;1 expression in *Pichia pastoris* cells

Competent X-33 *P. pastoris* cells (Invitrogen) transformed with the linearized *HvNIP2;1*-Myc-6xHis-pPICZ-B were streaked on the YPD plates (composition defined in the EasySelect Pichia Expression Kit Manual) containing 100 μg/ml zeocin (InvivoGen) and incubated for 2 days at 28 °C (3). Cells from a single colony were inoculated into 2 mL of liquid BMGY media (composition defined in the EasySelect Pichia Expression Kit Manual) in 10 ml conical test tubes. Liquid cultures were grown for 2 days at 25 °C, transferred to 200 ml of liquid BMMY media (composition defined in the EasySelect Pichia Expression Kit Manual), and induced with 1% (v/v) methanol in 2-L Erlenmeyer flasks for 4 days at 25 °C maintaining 1% (v/v) methanol under shaking (120 rpm; Multitron INFORS HT). Cells were harvested by centrifugation (4500*g*, 10 min, ambient temperature), pellets resuspended in 10% (v/v) glycerol, and stored at −80 °C.

### Purification of HvNIP2;1 *via* Immobilized Metal Affinity Chromatography (IMAC)

The SMA-solubilized preparation (49) was incubated with 0.5 to 1 ml of the Complete His-Tag Purification Resin (Roche, Indianapolis, IN, USA) equilibrated in SB and incubated for 16 to 18 h at ambient temperature. Resin with bound protein was packed in a disposable Bio-Rad column, and bound protein was eluted with 300 mM imidazole in SB at 1 ml/min flow rate at 4 °C (11). Fractions (1 ml) were analysed by SDS-PAGE combined with IB, as described above. Positive fractions were pooled and concentrated to 200 μl on a Microcon Ultracel YM10 micro-concentrator (50 kDa exclusion limit, Millipore Billerica). The final preparation was aliquoted and stored with 20% (v/v) glycerol at −80 °C.

### Reconstitution of HvNIP2;1 in liposomes

DMPC lipids were dissolved at 10 mg/ml in chloroform, dried on a rotary evaporator under vacuum for 30 min, and rehydrated in the liposome buffer (LB) [20 mM Tris-HCl (pH 8.0) containing 100 mM KCl]. The lipid mixture was sonicated until clear and filtered through 100 nm pores (Avanti) of a uniform size using the LiposoFast Hand Extruder (Avestin). The SMA-solubilized HvNIP2;1 protein preparation and filtered DMPC liposomes were mixed at a ratio of 1:50 on a weight basis, mixed by gentle shaking at room temperatures for 15 min and the mixture was dialyzed in LB containing 50 mM MgCl_2_ to disrupt the SMA polymer. The 50 mM MgCl_2_ concentration was maintained in all the buffers after this step. No protein precipitation was observed during the reconstitution of HvNIP2;1 in DMPC liposomes.

### Isolation of homogenous DMPC liposomes with reconstituted HvNIP2;1 through floatation on the iohexol gradient

Equal volumes (50 μl) of liposomes with reconstituted HvNIP2;1 and 80% (w/v) iohexol in the 25 mM HEPES-NaOH buffer (pH 7.5) containing 100 mM NaCl and 10% (w/v) glycerol were mixed (50, 51). The mixture was transferred to an ultra-clear polyallomer test tube (Beckman Coulter), overlaid with 350 μl of 30% (w/v) iohexol in the 25 mM HEPES-NaOH buffer (pH 7.5) containing 100 mM NaCl and 10% (w/v) glycerol, and with 100 μl of the 25 mM HEPES-NaOH buffer (pH 7.5) containing 100 mM NaCl. The mixture was centrifuged (100,000*g*, 4 h, four °C) in the L-80XP ultra-centrifuge using the SW55Ti swinging-bucket rotor (Beckman Coulter). After ultra-centrifugation, 60 μl fractions were sequentially collected from the top of the gradient to the bottom and examined by SDS-PAGE and IB. Selected fractions of liposomes with embedded HvNIP2;1 were pooled, centrifuged (10,000*g*, 2 min, 4 °C), resuspended in LB, dialyzed for 18 h in LB at 4 °C using 10-kD cut-off Slide-Alizarine dialysis cups (Thermo Fischer Scientific) and used in stopped-flow light scattering recordings. Fractionated proteo-liposomes stored on crushed ice remained stable for up to 5 days.

### Stopped-flow light scattering recordings of solute transport in proteo-liposomes with HvNIP2;1

Permeability of DMPC proteo-liposomes with reconstituted HvNIP2;1 and control liposomes lacking HvNIP2;1 was measured (46) to test the transport of 11 solutes with the DX.17 MV stopped-flow spectrophotometer (Applied Photophysics). The shrinking and re-swelling of vesicles were measured by 90° light scattering (550 nm) at 21 °C, upon rapidly mixing solutions that create an outward-directed concentration gradient with test solutions in the liposome buffer [20 mM Tris-HCl (pH 8.0) containing 100 mM KCl] at the concentration of 0.2 M (340 mOsmol/kg). These measurements were repeated and extended, such that in total, the transport of a panel of 27 solutes was investigated. Traces from five individual stopped-flow acquisitions were averaged and normalized shrinking and swelling kinetics were fitted to a non-linear regression single exponential function, from which rate constants were calculated. To inhibit solute permeation, the thiol-group modifier AgNO_3_ at 0.5 mM was used (52). Stopped-flow light scattering data were analyzed using Prism 9.0.0 (GraphPad Prism Software, Inc) based on two biological and two technical replicates of five averaged stopped-flow acquisitions, using non-linear regression of a one-phase decay (solutes) or two-phase association (water) models. Osmotic permeability *P* coefficients for water were calculated based on *P*_*water*_ = (V/A) × rate constant/(V_w_ × C_o_) (77) and permeability coefficients of all other solutes based on *P*_*solutes*_ = (V/A) × rate constant (78), where V/A is the volume to surface area ratio of liposomes, V_w_ is the partial molar volume of water and C_o_ is the external osmolarity after mixing, using the 50-nm liposome radius. *P*_*solutes*_ coefficients were corrected for nonselective diffusion through lipid bilayers using control liposomes. Diameters of DMPC liposomes with reconstituted HvNIP2;1 and control liposomes were determined using the NICOMP 380 Particle Sizing System operating in a vesicle mode. The data were weighted on ±60 liposomes.

### Heterologous expression of HvNIP2;1 in *X. laevis* oocytes and oocyte swelling

HvNIP2;1 expression in oocytes was performed as described (46, 47). Briefly, native HvNIP2;1 DNA was inserted in the Gateway-enabled pGEMHE vector (9, 79) and complementary RNA (cRNA) was transcribed using the Ambion mMESSAGE mMACHINE kit (Life Technologies). 23 ng cRNA in 46 nl of RNA-free water or an equal volume of RNA-free water were injected in oocytes, followed by incubation in ND-96 for 24 to 48 h before measurements (9, 79). Permeability of HvNIP2;1 expressed in oocytes to solutes was investigated after the transfer of oocytes to the 5-fold diluted solution of ND96 (5 mM HEPES-NaOH buffer, pH 7.4 containing 96 mM NaCl, 2 mM KCl, 1.8 mM CaCl_2_, and 1 mM MgCl_2_) supplemented with solutes at 160 mM concentration, which equaled to 200 mOsmol/kg osmolarity. 0.5 mM H[AuCl_4_] (53) was used to inhibit the permeation of HvNIP2;1 expressed in oocytes.

### 3D molecular model of HvNIP2;1, crystal structure of SoPIP2;1, and sucrose docking and evaluations of permeation tunnels

The coordinates of a monomeric 3D HvNIP2;1 model from barley (*H. vulgare* L.) were taken from the AlphaFold Protein Structure Database (54; accession AF-D8V828-F1). In this AlphaFold HvNIP2;1 model, six residues were positioned in disallowed regions, which were all positioned at the N-terminal loop (A2, S3, N8, R10, S15, and E16) and which was not used in docking or structural evaluations of permeation pores.

The coordinates of spinach (*Spinacia oleracea* L.) SoPIP2;1 (PDB accession 2B5F) in an open state conformation (38, 39) and those of Lsi1 (PDB accession 7cjs) (42) OsNIP2;1 (PDB accession 7nl4) (43) and were taken from the Protein Data Bank.

The root-mean-square deviation values of 0.67 Å and 0.45 Å, between the HvNIP2;1 AlphaFold model and OsNIP2;1 or Lsi1, indicated high similarity at structural levels of all three AQPs, which was underlined by high sequence identities amongst three AQPs (defined in the Results section).

Docking of sucrose into HvNIP2;1 and SoPIP2;1 was performed using the HDOCK server (58), which is based on a hybrid algorithm of template-based modeling and *ab initio*-free docking of protein-protein and protein-ligand/DNA/RNA/systems through generating up to 100 docking poses. With each AQP, all 100 models were evaluated, and the top five models were selected (based on the position of sucrose within permeating pores) and used for analyses of sucrose dispositions.

Permeation tunnels in HvNIP2;1 and SoPIP2;1 and their properties such as lengths and diameters, were evaluated by Caver v.3.0.3 (56) (with maximum distance and desired radius parameters of 3 Å and 5 Å, respectively), embedded in the PyMol v.2.5.4 software (Schrődinger LLC), which was also used for the generation of graphics images of HvNIP2;1 and SoPIP2;1.

### Phylogenomic analyses

Viridiplantae MIP sequences with matches to the PF00230 PFAM (80) were retrieved from Phytozome 12.1 (https://phytozome.jgi.doe.gov/pz/portal.html) (81) (Dataset S1). Archaean, bacterial, fungal, and metazoan sequences were retrieved from the UniProt reference genomes using top hits from the EBI hmmsearch implementation (https://www.ebi.ac.uk/Tools/hmmer/search/hmmsearch) (82, 83). Barley and wheat sequences were curated from in-house collections and the NCBI GenBank (84). Excessively long, short, or fragmented sequences were manually removed. Two datasets were prepared, one with only Viridiplantae and a larger expanded collection of plant and archaean, bacterial, fungal, and metazoan sequences. Jalview (85) was used to identify a 90% redundancy threshold for archaean, bacterial, fungal, and metazoan sequences and to manually select cluster representatives. The AlignSeqs function from DECIPHER (81) was used to align 164 selected Viridiplantae sequences. Clipkit (86) was used to trim excessively gapped sites under the gap model (g = 0.95). The expanded all domain dataset was prepared using hmmalign where residues were assigned to the MIP PF00230 HMM from the Pfam database. The flanking unassigned residues were excluded using the him malign trim function.

As MIP sequences were relatively short, data could not support the fit for complex, parameter-rich models to deep alignments. Thus, maximum-likelihood analyses were restricted to Viridiplantae, while the analyses of Virdiplantae, archaean, bacterial, fungal, and metazoan sequences were limited to distance methods. Substitution model selection for the Viridiplantae and all domain data was performed using ModelTest-NG (87) with LG+G4 determined as best fit for both data under the AICc. The phylogeny was calculated using RAxML-NG v1.0.2 (88). The best-known maximum-likelihood tree was selected based on final GAMMA scores after 150 random and 150 parsimony start-tree searches. Confidence values were determined by calculating 1000 transfer bootstrap estimate replicates (89). Distance analysis was performed with FastME 2.0 (90) using the LG+G4 substitution model. Confidence values were determined by calculating 1000 bootstrap replicates.

## Data availability

All data are included within the manuscript and its Supporting information.

## Supporting information

This article contains Supporting information (10, 46, 47, 48, 49, 51, 92, 93, 94, 95) .

## Conflict of interest

The authors declare that they have no conflicts of interest with the contents of this article.
