# Supplementary material for: Barley Nodulin 26-like intrinsic protein permeates water, metalloids, saccharides, and ion pairs due to structural plasticity and diversification
Source: J Biol Chem. 2023 Oct 31;299(12):105410. doi: 10.1016/j.jbc.2023.105410 (PMC10716587; doi:10.1016/j.jbc.2023.105410)
Supplement: Supplemental Dataset S1 Legend, Figures S1–S3, and Tables S1 and S2 [file mmc1.pdf]

Supplementary Information

Barley Nodulin 26-like Intrinsic Protein permeates water, metalloids, saccharides, and ion pairs due to structural plasticity and diversification

Akshayaa Venkataraghavan[1], Julian G. Schwerdt[1], Stephen D. Tyerman, and Maria Hrmova[2]

School of Agriculture, Food and Wine, and Waite Research Institute, University of Adelaide, Waite Research Precinct, Glen Osmond, SA, Australia

[1] A.V. and J.G.S. contributed equally to this work.
[2] To whom correspondence may be addressed. Email: maria.hrmova@adelaide.edu.au.

ORCID: 0009-0001-4572-0656 (A.V.); 0000-0002-0476-031X (J.G.S.); 0000-0003-2455-1643 (S.T.D.); 0000-0002-3545-0605 (M.H.).

This PDF file includes:

- Supplementary experimental procedures
- Supplementary references
- Legend for Supplementary dataset S1
- Supplementary figures S1-S3
- Supplementary tables S1-S2

Other supplementary materials include:

- Supplementary dataset S1

The experiments presented in the Supporting Information did not require new procedures or supporting material compared to the main text.

Supplementary experimental procedures

Materials

Oligonucleotide primers, restriction enzymes, plasmid extraction kits, the EDTA-free Complete Protease Inhibitor Cocktail, iohexol (Accudenz), Blue Dextran, and other chemicals were sourced as described (1-4). Benzonase (40 U/ml) was purchased from Invitrogen (Carlsbad, CA, USA), 1,2-dimyristoyl-sn-glycero-3-phosphocholine (DMPC) was from Avanti Polar Lipids (Alabaster, AL, USA), and the styrene-maleic anhydride co-polymer 3:1 (SMA) was provided by the courtesy of Dr. Timothy J. Knowles (University of Birmingham, United Kingdom).

Fractionation of Pichia pastoris cells using urea/alkali treatment

The distribution of HvNIP2;1 in *Pichia* cellular fractions was evaluated as follows (4). In brief, cells were lysed in the 90% (v/v) Yeast-Buster reagent (Invitrogen), 1% (v/v) benzonase (Invitrogen), 2 mM mercaptoethanol, 10 mM EDTA, 1 mM PMSF, and an EDTA-free Complete Protease Inhibitor Cocktail tablet (Roche, Indianapolis, IN, USA). The mixture incubated at ambient temperature for 20 min on a microtiter shaker at 300 rpm (Ratek, Victoria, Australia) was centrifuged (4,500xg, 10 min, ambient temperature) to yield the first soluble fraction. The pellet was resuspended in 4% (w/v) sodium dodecyl sulphate (SDS), and after incubation (10 min, ambient temperature) the second solubilised fraction was collected (4,500xg, 10 min, ambient temperature). The pellet was solubilised in 8 M urea in 0.1 M NaOH to collect the third solubilised fraction after centrifugation (4,500xg, 10 min, ambient temperature). The fourth fraction was an insoluble material after alkali/urea treatment. Fractions 1-4 were evaluated by the immunoblot blot analyses using a mouse anti 6xHis monoclonal (Clontech, Takara Bio, Shiga, Japan) and polyclonal antibody raised against HvNIP2;1 (using the SVAADDDELDHIPV C-terminal peptide) (5), provided by the courtesy of Professor Jian Feng Ma (Okayama University, Japan). Samples containing high levels of HvNIP2;1 were analysed by SDS-PAGE and immunoblot (IB) analyses.

Enzymatic digestion and disruption of Pichia cells to isolate microsomal membrane fraction

Pichia cells with the highest expression of HvNIP2;1, based on the fractionation technique described above (4) were selected. Cells grown as described above were thawed on ice and digested with Zymolyase from *Arthrobacter luteus* (MPI Biomedicals, Australia) at 1 U/g of cells (6) in the 10 mM Tris-HCl buffer (pH 6.5) under rotary shaking for 2 hours at ambient temperature. Digested cells were centrifuged (500xg, 10 min, 4 ºC), and the pellet was re-suspended in a breaking buffer (BB) [50 mM sodium phosphate buffer (pH 7.5), 5% (v/v) glycerol, 1 mM DTT, 1 mM PMSF and 1 mM EDTA] and disrupted in a BeadBeater Disrupter (Biospec Products, Bartlesville, OK, USA) with acid-washed glass beads of 0.1 mm diameter (Biospec Products) using the protocol as described (7). Briefly, cells were processed *via* a ten-times disruption protocol (each lasting for 1 min), with intermittent cooling intervals (3 min), disintegrated cells centrifuged (12,000xg, 10 min, 4 ºC) and the cell debris and glass beads were suspended in 1 mL of BB, vortexed and centrifuged (12,000xg, 10 min, 4 ºC). The supernatant was transferred to polyallomer test tubes (Beckman Coulter, CA, USA) and subjected to high-speed centrifugation (200,000xg, 60 min, 4 ºC). The pellet containing the microsomal membrane fraction was resuspended in 100 µL of a storage buffer [50 mM sodium phosphate buffer (pH 7.5) 10% (v/v) glycerol, 1 mM DTT, 1 mM

PMSF, 0.1 mM EDTA, 100 mM KCl, and an EDTA-free protease inhibitor cocktail (Roche)] and evaluated for the presence of HvNIP2;1 by SDS-PAGE combined with IB analyses. Alternatively, the membrane pellet was processed as described in the section below.

Urea/alkali treatment of microsomal membrane fractions

The membrane pellet collected by ultra-centrifugation from the last step described in the above section was treated as follows (7, 8): the pellet was resuspended in the 5 mM Tris-HCl (pH 9.5) buffer, containing 5 mM EDTA, 5 mM EGTA and 4 M urea, and contaminating proteins adhering to membranes were removed by centrifugation (100,000g, 40 min, 4 ^{o}C). The pellet was suspended in 20 mM NaOH (approximate pH 12), incubated for 5 min at ambient temperature, centrifuged (100,000xg, 40 min, 4 ^{o}C) and re-suspended in the 5 mM Tris-HCl (pH 8) buffer containing 2 mM EDTA, 2 mM EGTA, 100 mM NaCl, and again cleared by centrifugation (100,000xg, 40 min, 4 ^{o}C). The final pellet containing urea/alkali-stripped microsomal plasma membranes were suspended in 100-250 µL (depending on the amounts of used cells) in the 20 mM HEPES-NaOH (pH 7.8) solubilisation buffer (SB) containing 50 mM NaCl, 10% (v/v) glycerol, 2 mM mercaptoethanol, and stored on ice to proceed with SMA solubilisation.

Solubilisation of HvNIP2;1 from the urea/alkali-treated microsomal membrane fractions by SMA

This step was conducted on the urea/alkali-treated microsomal membrane fraction obtained as described above, by adding SMA (9) from the 10% (w/v) stock solution in SB to 2% (w/v) concentration. Solubilisation proceeded for 4 hours under rotary shaking, after which the solubilised protein was centrifuged (200,000xg, 60 min, 4 °C) and the supernatant fraction was used for purification of the HvNIP2;1 protein. No additional SMA was added during further purification steps.

Analytical techniques

Protein samples were mixed with the SDS-PAGE loading buffer (10) and incubated at 37 ^{o}C for 30 min. Samples are loaded onto a gel and run at 150 V for 90 min followed by IB analyses using 0.45 µm polyvinyl difluoride transfer membrane and detection with antibodies. Working solutions of antibodies were prepared in 25 mM Tris-HCl buffer, pH 7.5 containing 137 mM NaCl, 3 mM KCl, and 0.05% (w/v) Tween 20. Blots were incubated with antibodies for 1-16 hours with gentle agitation at 4-8 ^{o}C. IB blots were developed with the Novex® ECL HRP Chemi-Luminescent Substrate Reagent Kit (Invitrogen) or with the BCIP/NBT-purple liquid reagent (Sigma-Aldrich) following the manufacturer's instructions. Proteins on SDS-PAGE gels were stained with Coomassie Brilliant Blue R-250 (Sigma-Aldrich) and the semi-quantitative estimation of protein content on SDS-PAGE gels was based on known amounts of BSA (fraction V, Sigma-Aldrich).

Supplementary references

1. Rongala, J. (2016) Characterisation of cereal transport proteins involved in boron toxicity tolerance. Ph.D. thesis, The University of Adelaide. Published by The University of Adelaide, Adelaide, Australia

2. Venkataraghavan, A. (2017) Transport characteristics of a plant aquaporin from the Nodulin-26 like intrinsic protein (NIP) family. MSc thesis, SASTRA (Shanmugha Arts Science Technology & Research Academy) University and The University of Adelaide. Published by The University of Adelaide, Adelaide, Australia

3. Nagarajan, Y. (2010) A silicon transporter involved in abiotic stress tolerance in cereal plants: molecular cloning and recombinant protein expression. MSc thesis, Flinders University and the University of Adelaide. Published by The University of Adelaide, Adelaide, Australia

4. Nagarajan, Y., Rongala, J., Luang, S., Singh, A., Shadiac, N., Hayes, J., Sutton, T., Gilliham, M., Tyerman, S. D., McPhee, G., Voelcker, N. H., Mertens, H. D. T., Kirby, N. M., Lee, J.-G., Yingling, Y. G., and Hrmova, M. (2016) A barley efflux transporter operates in a Na^+-dependent manner, as revealed by a multidisciplinary platform. *Plant Cell* **28**, 202–218

5. Chiba, Y., Mitani, N., Yamaji, N., and Ma, J. F. (2009) HvLsi1 is a silicon influx transporter in barley. *Plant J*. **57**, 810–818

6. Kaneko, T., Kitamura, K., and Yamamoto, Y. (1973) Susceptibilities of yeasts to yeast cell wall lytic enzyme of Arthrobaeter luteus. *Agric. Biol. Chem.* **37**, 2295–2302

7. Hasler, L., Walz, T., Tittmann, P., Gross, H., Kistler, J., and Engel, A. (1998) Purified lens major intrinsic protein (MIP) forms highly ordered tetragonal two-dimensional arrays by reconstitution. *J. Mol. Biol.* **279**, 855–864

8. Karlsson, M., Fotiadis, D., Sjövall, S., Johansson, I., Hedfalk, K., Engel, A., and Kjellbom, P. (2003) Reconstitution of water channel function of an aquaporin overexpressed and purified from Pichia pastoris. *FEBS Lett*. **537**, 68–72

9. Knowles, T. J., Finka, R., Smith, C., Lin, Y.-P., Dafforn, T., and Overduin, M. (2009) Membrane proteins solubilized intact in lipid containing nanoparticles bounded by styrene maleic acid copolymer. *J. Am. Chem. Soc.* **131**, 7484–7485

10. Luang, S., Hrmova, M., and Ketudat Cairns, J. R. (2010) High-level expression of barley beta-D-glucan exohydrolase HvExoI from a codon-optimized cDNA in Pichia pastoris. *Prot. Expr. Purif*. **73**, 90–98

Legend for Supplementary dataset S1

List of 3,157 Viridiplantae, archaean, bacterial, fungal, and metazoan sequences investigated in this work.

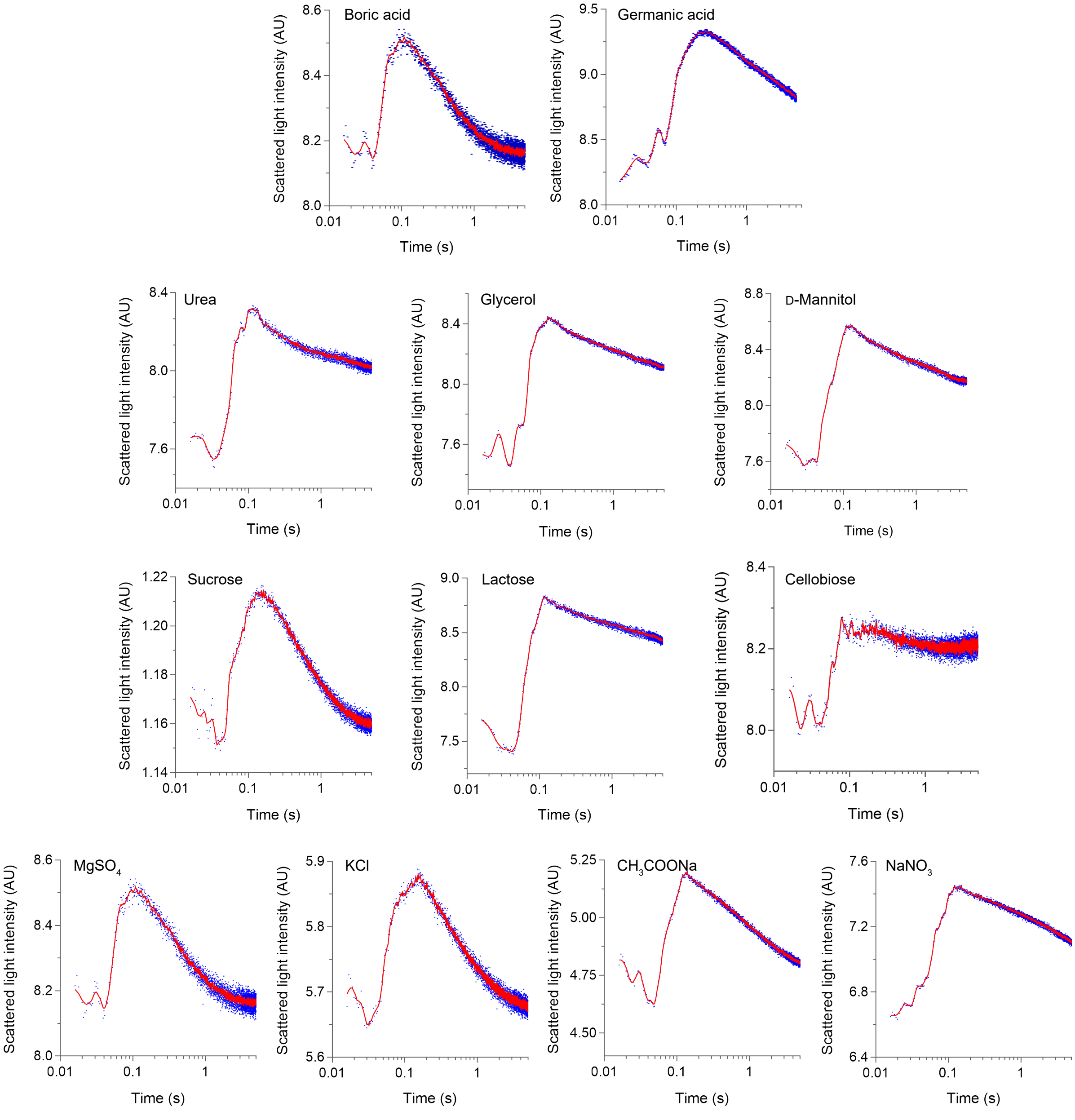

Figure S1. Transport of permeants by HvNIP2;1 embedded in liposomes.

DMPC liposomes with embedded HvNIP2;1 were exposed to gradients of permeants generating osmotic gradients with BA and germanic acid (top panels), urea, glycerol, and D-mannitol (top-middle panels), disaccharides sucrose and lactose and a monosaccharide L-arabinofuranose (bottom-middle panels) and $MgSO_4$, KCl, CH_3COONa and $NaNO_3$ (bottom panels). Uptake of permeants was measured by stopped-flow spectrophotometry, as described in Fig. 2, where light scattering due to the initial vesicle shrinkage (initial peak) and subsequent swelling (slope) were monitored for five seconds. Light scattering traces of five averaged traces of each permeant representing the raw (blue trace) and smoothed (red trace) data were plotted in arbitrary units (AU) in GraphPad Prism 9.

Table S1. Rate constants and permeability coefficients of barley HvNIP2;1 embedded in liposomes [a]

Permeant [b]	Structure	Rate constant (s^{-1}) [c]	*P* coefficient (cm s^{-1})
**Solute**			
Water [d]	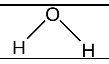	73.15±3.04	3.98×10^{-2}±16.0
Boric acid	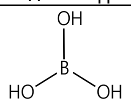	1.75±0.02	2.50×10^{-6}±0.03
Germanic acid	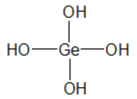	0.59±0.02	0.94×10^{-6}±0.03
Urea	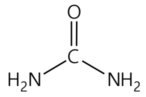	0.95±0.01	1.49×10^{-6}±0.02
Glycerol	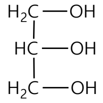	0.88±0.01	1.44×10^{-6}±0.02
D-Mannitol	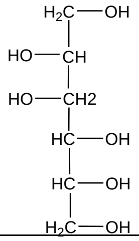	0.77±0.02	1.25×10^{-6}±0.03
**Mono- and disaccharides**			
Sucrose	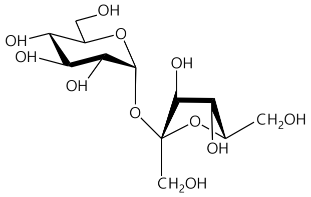	1.31±0.01	1.58×10^{-6}±0.02
Lactose	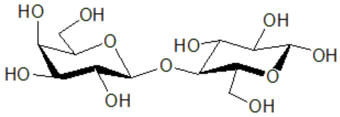	0.70±0.01	0.73×10^{-6}±0.13
L-Arabinofuranose	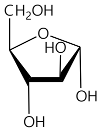	0.36±0.10	0.23×10^{-6}±0.26
**Ions**			
$MgSO_4$	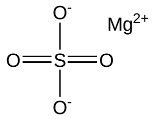	1.75±0.02	2.66×10^{-6}±0.03
KCl	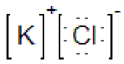	1.33±0.02	1.54×10^{-6}±0.03
CH_3COONa	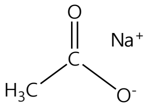	0.79±0.03	1.06×10^{-6}±0.06
$NaNO_3$	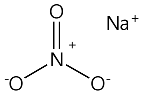	0.46±0.05	0.30×10^{-6}±0.08

[a] SMA-solubilised barley HvNIP2;1 was reconstituted in DMPC liposomes.

[b] Permeability coefficients of proteo-liposomes with HvNIP2;1 and control liposomes for water were calculated using P_{water} = (V/A) × rate constant / (V_w × C_o), and those for other solutes P_{solute} = (V/A) × rate constant, where V/A is the volume to surface area ratio of liposomes (radius 50-nm), V_w is the partial molar volume of water and C_o is the external osmolarity after mixing. Values were rounded to two decimal points.

[c] Calculated with GraphPad Prism 9 based on two biological and two technical replicates of five averaged stopped-flow acquisitions.

[d] The P_{water} coefficients ratio of proteo-liposomes with HvNIP2;1 (3.98×10^{-2} cm s^{-1}) *versus* empty liposomes (1.66×10^{-4} cm s^{-1}) is 240.

Table S2. Distribution of peptide motifs in bipartite segments of barley HvNIP2;1

Sequence position	P-value [a]	1st Repeat	Structural element	Sequence position	P-value [a]	2nd Repeat	Structural element	Colour in image
31-37	2.5e-09	MVYYTER	N-terminal loop	252-258	7.8e-09	WTYTYIR	α-helix 6	Yellow
38-43	4.9e-07	SIADYF	N-terminal loop	116-121	8.8e-08	AIFRHF	H3-HB loop	Forest green
44-51	1.1e-08	PPHLLKK	α-helix 1	263-269	5.4e-10	PKDAPQK	C-terminal loop	Cyan
54-66	2.1e-14	EVVSTFLL VFVTC	α-helix 1	170-182	2.9e-15	EVVVTFNM MFVTL	α-helix 4	Magenta
74-79	5.4e-08	HDVTRI	Loop A	149-154	2.2e-07	HPITVI	Loop C	Blue
139-144	9.2e-07	CASFVL	α-helix 3	204-209	1.9e-07	CITSIF	α-helix 5	Red
95-103	4.7e-08	MIYAVGH	α-helix 2	187-193	7.0e-09	DTRAVGE	Loop D	Black
107-112	4.6e-08	M**NPA**VT [b]	Re-entrant α-helix HB	218-223	1.0e-08	M**NPAR** [b] ***T*** [c]	Re-entrant α-helix HE	Green
123-130	4.9e-07	WIQVPFY W	α-helix 3	230-237	8.8e-08	SNRYPGLW	α-helix 6	Grey

[a] P-value is a measure of the false discovery rate of each analysed match (57).

[b] NPA and R222 selectivity filter residues are in underlined bold or bold, respectively.

[c] Froger's P2 T223 position is in italics bold. P1 (L148) and P3-P5 (A227, Y239, F240) are excluded.

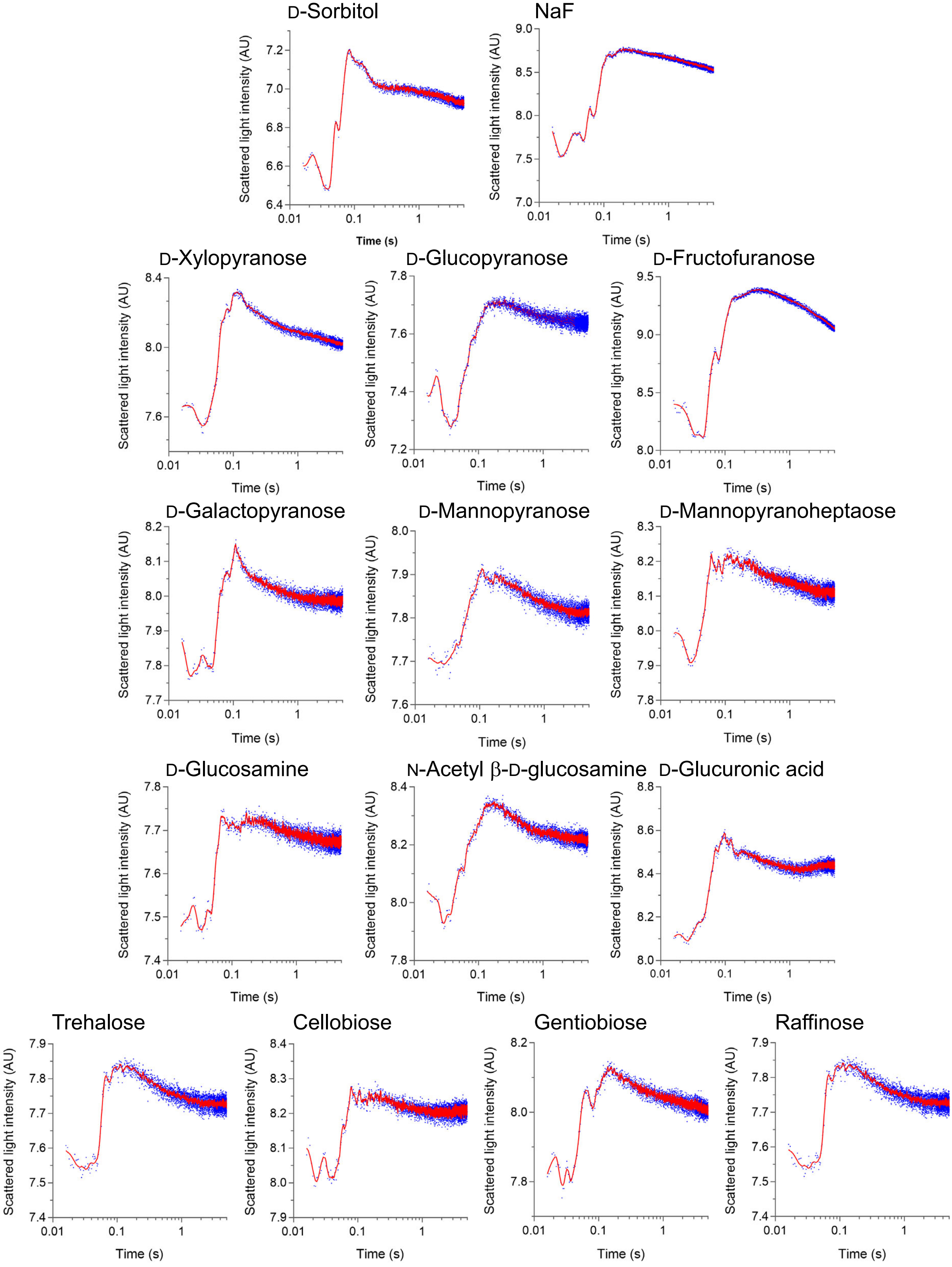

D-Sorbitol
NaF
D-Xylopyranose
D-Glucopyranose
D-Fructofuranose
D-Galactopyranose
D-Mannopyranose
D-Mannopyranoheptaose
D-Glucosamine
N-Acetyl β-D-glucosamine
D-Glucuronic acid
Trehalose
Cellobiose
Gentiobiose
Raffinose
Scattered light intensity (AU)
Time (s)

Figure S2. Transport of permeants by HvNIP2;1 embedded in liposomes.

DMPC liposomes with embedded HvNIP2;1 were exposed to gradientso gradients of solutes generating osmotic gradients with D-sorbitol, NaF (top panels), monosaccharides D-xylopyranose, D-glucopyranose, D-fructofuranose, D-galactopyranose, D-mannopyranose and D-mannopyrano-heptaose (two top-middle panels), derivatised monosaccharides D-glucosamine, N-acetyl β-D-glucosamine, and D-glucuronic acid (bottom-middle panel), and disaccharides trehalose, cellobiose, gentiobiose, and trisaccharide raffinose (bottom panels). The uptake of permeants was measured by stopped-flow spectrophotometry, as described in Fig. 2. Light scattering traces of five averaged traces for each permeant representing raw (blue trace) and smoothed (red trace) data were plotted in arbitrary units (AU) in GraphPad Prism 9.

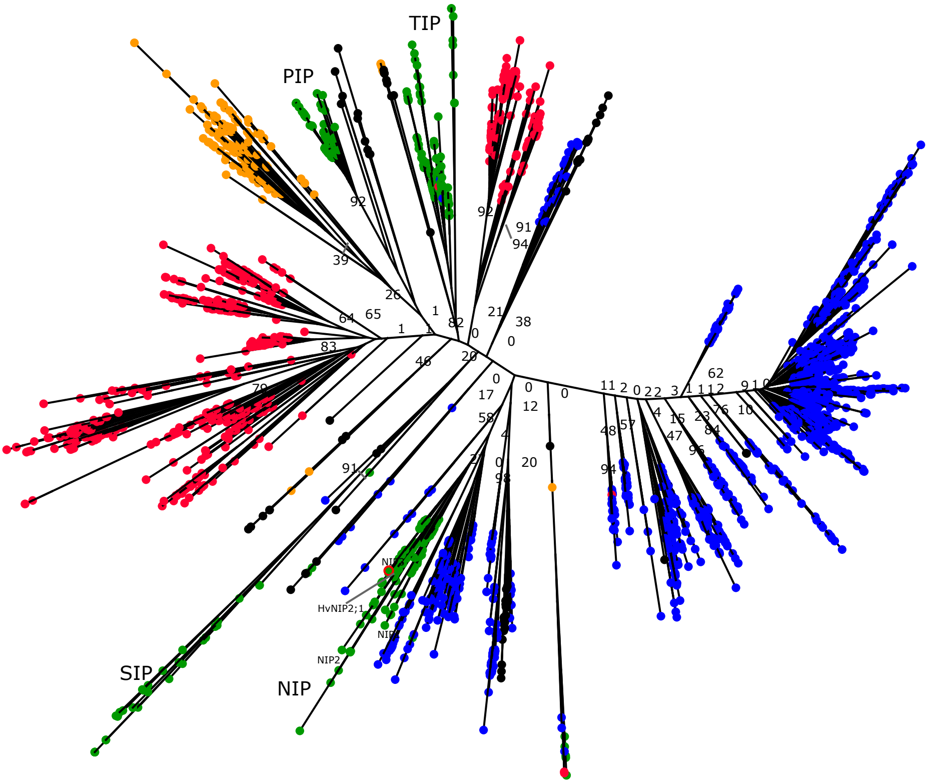

Figure S3. FastME distance tree of 3,157 PF00230 MIP proteins.

Terminal nodes are colour-coded by kingdom: green, Viridiplantae; black, Archaea; blue, Bacteria; yellow, Fungi; red, Metazoa. The list of individual 3,157 entries is specified in Dataset S1. Bootstrap support values are indicated at major nodes.
